# Development of a novel mobile application, HBB Prompt, with human factors and user-centred design for Helping Babies Breathe skills retention in Uganda

**DOI:** 10.1186/s12911-021-01406-z

**Published:** 2021-02-04

**Authors:** Natalie Hoi-Man Chan, Hasan S. Merali, Niraj Mistry, Ryan Kealey, Douglas M. Campbell, Shaun K. Morris, Santorino Data

**Affiliations:** 1 Division of Neonatology, British Columbia Women’s Hospital, 1N55-4480 Oak Street, Vancouver, BC V6H 3V4 Canada; 2 Division of Pediatric Emergency Medicine, Department of Pediatrics, McMaster Children’s Hospital, 1280 Main Street West, HSC-2R104, Hamilton, ON L8S 4K1 Canada; 3 Division of Paediatric Medicine, Department of Pediatrics, The Hospital for Sick Children, 555 University Avenue, Toronto, ON M5G 1X8 Canada; 4 Interactive Media Lab, University of Toronto, 5 King’s College Road, Toronto, ON M5S 3G8 Canada; 5 Design Research, TD Bank Group, Toronto, ON Canada; 6 Division of Neonatology, The Hospital for Sick Children, Toronto, ON Canada; 7 Neonatal Intensive Care Unit, St. Michael’s Hospital, 15014 - 30 Bond St, Toronto, M5B 1W8 ON Canada; 8 Division of Infectious Diseases and Centre for Global Child Health, The Hospital for Sick Children, 555 University Avenue, Toronto, ON M5G 1X8 Canada; 9 Department of Pediatrics, University of Toronto, Toronto, ON Canada; 10 Department of Pediatrics and Child Health, Mbarara University of Science and Technology, P.O. Box 1410, Mbarara, Uganda; 11 Consortium for Affordable Medical Technologies in Uganda (CAMTech Uganda), Mbarara, Uganda

**Keywords:** Newborn resuscitation, Helping babies breathe, Mobile application, Uganda, Simulation, Human factors, User-centred design, mHealth

## Abstract

**Background:**

Helping Babies Breathe (HBB) is a life-saving program that has helped reduce neonatal morbidity and mortality, but knowledge and skills retention after training remains a significant challenge for sustainability of impact. User-centred design (UCD) can be used to develop solutions to target knowledge and skills maintenance.

**Methods:**

We applied a process of UCD beginning with understanding the facilitators of, and barriers to, learning and retaining HBB knowledge and skills. HBB Master Trainers and frontline HBB providers participated in a series of focus group discussions (FGDs) to uncover the processes of skills acquisition and maintenance to develop a mobile application called “HBB Prompt”. Themes derived from each FGD were identified and implications for development of the HBB Prompt app were explored, including feasibility of incorporating strategies into the format of an app. Data analysis took place after each iteration in Phase 1 to incorporate feedback and improve subsequent versions of HBB Prompt.

**Results:**

Six HBB trainers and seven frontline HBB providers participated in a series of FGDs in Phase 1 of this study. Common themes included lack of motivation to practise, improving confidence in ventilation skills, ability to achieve the Golden Minute, fear of forgetting knowledge or skills, importance of feedback, and peer-to-peer learning. Themes identified that were not feasible to address pertained to health system challenges. Feedback about HBB Prompt was generally positive. Based on initial and iterative feedback, HBB Prompt was created with four primary functions: Training Mode, Simulation Mode, Quizzes, and Dashboard/Scoreboard.

**Conclusions:**

Developing HBB Prompt with UCD to help improve knowledge and skills retention was feasible and revealed key concepts, including drivers for successes and challenges faced for learning and maintaining HBB skills. HBB Prompt will be piloted in Phase 2 of this study, where knowledge and skills retention after HBB training will be compared between an intervention group with HBB Prompt and a control group without the app.

*Trial registration* Clinicaltrials.gov (NCT03577054). Retrospectively registered July 5, 2018, https://clinicaltrials.gov/ct2/show/study/NCT03577054.

## Background

Despite a significant reduction in global neonatal mortality from 1990 through 2018, approximately 2.5 million newborns die annually. These newborn deaths represent nearly half of the 5.3 million children under five who die each year [1]. The proportion of newborns who contribute to under-five mortality has been growing and the gap between reduction of childhood and infant mortality is rising. This is because strategies for newborn mortality reduction are challenging to implement [2]. The top three causes of newborn deaths continue to be prematurity, intrapartum-related events, and infection [2]. Approximately 10% of babies require help after birth in high-resource settings, compared with 15% in low-resource settings [3, 4]. Skilled personnel who can resuscitate babies immediately after delivery save lives [5, 6]. Standardized training through the Helping Babies Breathe (HBB) program has been shown to improve newborn resuscitation skills [7, 8].

The HBB curriculum, currently in its second edition (HBB 2.0), was designed to train frontline health providers in low-resource settings to provide life-saving interventions for newborns [9]. HBB has been taught in over 80 countries and has been shown to reduce neonatal mortality and morbidity [10]. HBB training materials are standardized with a flowchart that illustrates the steps in the HBB algorithm, accompanied by a facilitator flip chart that provides all the teaching prompts for HBB workshop delivery. The training program emphasizes the practice of individual steps, key skills, and integration of skills into the delivery setting through simulation. Illustrations and a green-yellow–red colour scheme are used to visually guide providers through the HBB algorithm. In Uganda, the HBB program has been deployed with the help of international health implementing partners in collaboration with the Ministry of Health due to the relatively high costs of HBB training [11]. One of the most essential skills HBB teaches is how to ventilate a newborn struggling to breathe, and to start doing so within one minute after birth, referred to as the Golden Minute. Skills deterioration after initial training is a significant barrier to sustained impact of programs like HBB [12]. Low dose high frequency (LDHF) practice, use of simulation, refresher training, and supportive supervision are strategies that help reduce the decay of skills over time [13–17]. However, implementing initial training, refresher training, and supportive supervision are all costly and resource intensive, particularly in settings where births occur at smaller facilities in rural communities spread out over a large geographic region [11]. Since 2010, the Ugandan Ministry of Health alongside implementing partners have been scaling up training for healthcare workers who look after newborns across the country using the HBB program. As of 2014, HBB was offered in more than 40% of health facilities [18].

Mobile health solutions have been increasingly popular as smart devices are ubiquitous. Their rate of ownership in low- and middle-income countries has grown exponentially, with over 5 billion unique users worldwide in 2019 [19], making it especially attractive when designing solutions aiming to reach large audiences [20]. In 2018, there were 19.8 million unique mobile phone users in Uganda, with half of these subscribers using the internet on their mobile phones [21]. This represents mobile internet penetration in 23% of the population. Of these users, only 16% had a smart phone compared with the average of 30% of users having smart phones in Sub-Saharan Africa [21]. Mobile applications have been tested in numerous settings within healthcare and medical education with varying success. Thoughtful development to optimize usability and utility for the target population is important [22–24]. Design processes with iterative prototyping have been successfully used to develop mobile apps for various purposes, such as supporting lifestyle interventions for cardiovascular health [25], clinical decision-making for osteoporosis treatment [26], and self-management for dementia patients [27]. Apps targeted towards newborn health [28–34] have been created and evaluated previously, some employing UCD, but none have informed their design by exploring the cognitive processes of learning newborn resuscitation skills. Understanding the underlying preferences, perceptions, facilitators and barriers that drive behaviours can help tailor the development of features and functions of apps [25, 35, 36]. Applying UCD and assessing human factors during the development of a mobile app can enhance user experience and increase the effectiveness of the tool.

In this paper we describe the UCD process of the development a mobile app (HBB Prompt) to improve knowledge and skills retention after initial HBB training.

## Methods

### Study overview

The study was conducted at Mbarara University of Science and Technology (MUST) in southwestern Uganda. This article describes Phase 1, the development of HBB Prompt. More details regarding the entire study can be found in the protocol paper [37]. Further details including the focus group discussion (FGD) guides for Phase 1 can be found in Additional file 1 and Additional file 2.

### Objectives

**Fig. 1:**
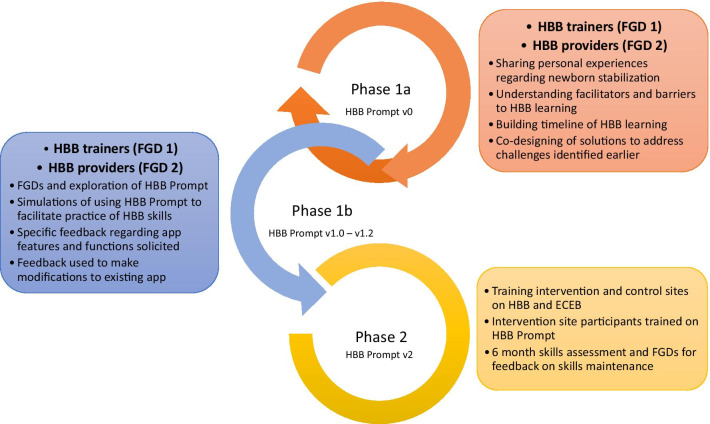
Overview of HBB Prompt study. The HBB Prompt study is divided into Phase 1, where there were iterative UCD cycles to develop HBB Prompt and Phase 2, where the HBB Prompt app will be piloted. During Phase 1, HBB trainers formed one focus group (FGD 1) and HBB providers formed the second focus group (FGD 2). Each group went through Phase 1a and 1b separately to facilitate UCD of HBB Prompt

The overall objectives of the study were to apply a UCD framework to develop a mobile app to enhance HBB knowledge and skills maintenance (Phase 1) and to test its effectiveness in a pilot setting (Phase 2). The objective of Phase 1a of the study was to understand the facilitators and barriers to learning and maintaining HBB skills for providers with varying levels of experience with newborn resuscitation. Information from Phase 1a was incorporated into modifications of HBB Prompt version 0 (v0) to create version 1 (v1), which was used in Phase 1b UCD testing. Subsequently, the objective for Phase 1b was to evaluate how HBB Prompt v1 does or does not meet the needs of HBB users and how it could be feasibly improved prior to pilot testing in Phase 2 of this study. See Fig. 1.

### Participant recruitment

Participants for Phase 1 were recruited by letter from a register of previously HBB-trained healthcare workers who reside and care for newborns in southwestern Uganda. Participants who responded and who were able to attend the scheduled FGD sessions were included in the study. Inclusion criteria for the first focus group was any healthcare provider with HBB trainer experience (i.e. received previous training in HBB and also completed the HBB Master Trainer Course) allowing them to teach the HBB curriculum. Inclusion criteria for the second focus group was frontline providers who had previously received HBB training.

### Phase 1: mobile app development

#### HBB Prompt v0 development

Study co-investigators collectively used their knowledge and experience with the HBB program, resuscitation education, and mobile app development to create foundational features of HBB Prompt v0. In particular, we sought to leverage strategies that have previously been shown to improve skills retention, including LDHF practice, use of simulation, refresher training, and supportive supervision [10, 38, 39]. This provided a framework for our two software developers to build features of the app that we anticipated HBB trainers and providers would request. The basic functions that were identified as most important were: training videos and information portal, self-practice mode and group simulation mode, knowledge assessments, and a practice dashboard.

The HBB Prompt app is an Android-based application that can run on smart devices with an Android Operating System, Version 4.0.3 and above. The Android platform was chosen due to lower cost and higher penetration of Android devices in Uganda and other low- and middle-income countries. The app was designed using Android Studio 3.0, an Integrated Development Environment provided by Google for design and development of Android apps. HBB Prompt, once installed, does not require internet connectivity; however, when internet connectivity is detected, it automatically logs usage data to a remote Linode server, which it maps according to device International Mobile Equipment Identity (IMEIs). Usage data tracked does not require runtime or dangerous permissions and therefore users do not need to be concerned about access to potentially sensitive data on their accounts. The app does not have access to audio or video recording, the camera function, or location data. Usage data recorded include user identification, date and time of login, time spent on each page of the app, and scores on quizzes or simulations. The app is designed to solely track in-app use, which is intended for education and practice only and not for direct patient care. The logged data can later be exported in comma-separated values (csv) format for further analysis.

#### Phase 1a

Two separate focus groups underwent the same sequence of exploratory activities: discussion of personal experiences with newborn resuscitation, facilitators and barriers to learning HBB knowledge and skills, perceptions of the timeline in acquisition of knowledge and skills, and generation of ideas on how to maintain and sustain HBB knowledge and skills. The composition of each focus group was primarily divided by HBB training experience so that input could be elicited separately from trainers (FGD 1) and frontline users (FGD 2) of the HBB curriculum. See Additional file 1 for details.

#### Phase 1b

The same focus groups were invited back to help co-design and provide feedback to improve the HBB Prompt v1. Participants explored the app, simulated use of the app and provided feedback regarding app content, interface, navigation, functionality, customizability, and usability. Two iterations of UCD feedback sessions took place prior to finalizing the app for Phase 2 pilot testing. See Additional file 2 for details.

## Analysis

FGDs were recorded and thematic analysis was conducted, where investigators independently coded components of the transcript to highlight needs and mental models of the participants. These independent codes were aggregated and synthesized in order to uncover main themes consistent across investigators. After each iteration, categories and themes were reviewed together with the software developers to incorporate feasible improvements to HBB Prompt.

## Results

### Participants

The first focus group comprised six HBB trainers who were all physicians and the second focus group comprised seven midwives. Table 1 describes the demographic characteristics of the participants in each FGD, including level of practical and simulated experience with newborn care, emergency training, and comfort with mobile devices.

**Table 1:** Focus group participant demographics and characteristics

	HBB trainers (n = 6)	HBB providers (n = 7)
Provider type	Medical Officers = 2 Medical Specialists = 4	Enrolled Midwife = 4 Registered Midwife = 3
Years of experience as healthcare provider	< 5 years = 1 5–10 years = 3 > 10 years = 2	< 5 years = 0 5–10 years = 4 > 10 years = 3
Age	21–30 years old = 1 31–40 years old = 5	21–30 years old = 1 31–40 years old = 5 41–50 years old = 1
Sex	Female = 4 Male = 2	Female = 7 Male = 0
*Health district*		
Ntungamo	1	0
Mbarara	5	7
*Level of Health facility*^a^		
Health Centre III	0	4
Health Centre IV	0	2
Hospital	6	1
*Training courses attended*		
Helping Babies Breathe (HBB)	6	7
Essential Care for Every Baby (ECEB)	6	5
Essential Care for Small Babies (ECSB)	4	4
Helping Mothers Survive—Bleeding after birth	2	1
Comprehensive Emergency Maternal and Newborn Care (CEMONC)	1	1
Basic Emergency Maternal and Newborn Care (BEMONC)	0	1
Emergency Triage and Treatment (ETAT)	4	1
Master Trainer for HBB	6	2
*Experience in last year*		
Number of births attended where babies not breathing at birth	None = 2 1–10 = 2 11–20 = 1 > 20 = 1	None = 0 1–10 = 4 11–20 = 1 > 20 = 2
Simulated deliveries with baby not breathing	None = 0 1–10 = 2 11–20 = 2 > 20 = 2	None = 0 1–10 = 4 11–20 = 2 > 20 = 1
Preterm deliveries attended	None = 1 1–10 = 2 11–20 = 2 > 20 = 1	None = 1 1–10 = 5 11–20 = 1 > 20 = 0
Simulated preterm deliveries	None = 2 1–10 = 3 11–20 = 0 > 20 = 1	None = 2 1–10 = 4 11–20 = 0 > 20 = 1
Deliveries attended in the last month^a^ (at the time of first FGD session)	None = 6	None = 1 1–10 = 2 11–20 = 3 > 20 = 1
Ownership of smart device	Yes = 6 No = 0	Yes = 6 No = 1
Comfort with using smart device (Likert scale 1–5)	Not comfortable = 0 Somewhat comfortable = 4 Very comfortable = 2	Not comfortable = 1 Somewhat comfortable = 5 Very comfortable = 1

^a^Health Centre III is a local clinic with access to delivery services and inpatient care, Health Centre IV is a small local hospital

### Phase 1a

#### Exploring personal experience, facilitators and barriers

Themes that emerged from FGDs are summarized in Table 2. Important factors that motivated HBB learning and skills maintenance were supportive supervision and self-esteem. Additionally, continuing medical education opportunities, peer-to-peer learning, refresher courses, and timely feedback were all identified as positive drivers. Barriers identified were lack of equipment and health system issues such as inadequate transport for escalation of care.

**Table 2:** Themes to address in HBB learning and skills maintenance during Phase 1a

Themes	Implications on app development	Selected quotes
*Facilitators*		
Peer-to-peer learning	Design of rater mode to support group practice through simulation Scoreboard/Dashboard feature to keep each other accountable for practice	“I see [peer learning] as helpful in the HBB training because most of us don’t go on the same pace. They sit in class together but then find that some pick faster and others nothing at all but when you are together in a health facility…we share what I did not get while someone did, so we learn from our friends”
Supportive supervision		“helpful when mentors use checklist while giving feedback—so every person knows where they made mistakes and where to keep learning”
Continuing Medical Education and Refresher courses	Repository of HBB information including demonstration videos	
Self-confidence	Scoreboard/Dashboard feature to track practice statistics and longitudinal trends of scores on quizzes	“I know I can do some good work and since then I have never lost any other baby and I am glad that HBB did some good work”
Timely feedback	Use of checklists to help review required steps in HBB after simulation completed Rater mode to help facilitate peer-to-peer feedback after simulation	“If [you] practice the same thing in and out on your own—may not be as effective as having someone to give you feedback, someone with more experience to give you more input on how to improve”
Safe learning environment	Use of audio and visual prompts to facilitate different ways of learning	
*Barriers*		
Inadequate or inappropriate resuscitation equipment for practice or for clinical use	Videos to review choice of and maintenance of resuscitation equipment	“We come here to do the training and practice but when we leave this place and go back to our health centres we don’t have what to use. You feel you have the skills, but you don’t have any equipment to use so some of the skills end up dying there.”
Lack of motivation to practice Too much self-pride Lack of support from colleagues to practise together, or to acknowledge and support the importance of practice	Scoreboard/Dashboard feature to track practice statistics and results of quizzes to encourage friendly competition and keep each other accountable Scoreboard/Dashboard to act as a reminder system to encourage ongoing practice	
Lack of confidence		
Infrequent trainings	Videos to review individual HBB skills Simulation mode to put knowledge and skills together and applying them to simulated delivery scenario	
Fear of not knowing how to translate skills from a training setting to real life scenarios	Design of simulation mode is to facilitate ongoing practice of skills to best mimic real-life resuscitations	

#### Timeline of learning HBB and perceptions of what was difficult to learn and remember

**Fig. 2:**
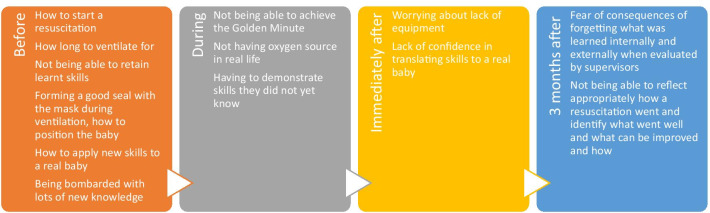
Insights about the timeline in HBB learning and skills maintenance during Phase 1a. Themes from FGDs with HBB trainers and providers regarding their thought process in relationship to the timing of HBB training

Participants explored the timeline of learning HBB skills and knowledge, including what was perceived as, and what was in reality, the most difficult to learn and remember (Fig. 2). A concern throughout was how to translate skills from a training environment to real-life scenarios. Before HBB training, many concerns surrounded learning about the steps to help a baby breathe and the fear of not retaining skills. During HBB training, there was fear of not achieving the Golden Minute. Three months after training, participants were worried about forgetting their skills and not effectively debriefing to improve after a resuscitation.

#### Co-designing solutions

Prior to being exposed to HBB Prompt v0, participants were asked to co-design solutions to help learn and maintain HBB skills. They identified promoting a safe learning environment as an important goal. To achieve this, suggestions emphasized the use of audio and visual prompts and written and video guides to facilitate different ways of learning. To counteract the inertia to practise, methods of motivating users were discussed, including setting reminders, promoting champions, having dedicated group practice sessions, and creating a friendly competitive environment to increase accountability. Participants also expressed the importance of a community of support, for example, through a message board or group chat to review cases and to encourage each other. Suggested mobile app features included information for each HBB skill (introduction, video demonstration, practice prompts, scenarios, and if possible, ability to provide feedback) and the ability to function offline as internet connectivity can often be unreliable.

#### Implications on app development

Themes that were not feasible to address using a mobile app included health system challenges such as lack of available equipment and transport for escalation of care for unstable babies. Additionally, due to limited funds to facilitate real-time internet connectivity of the app, the reminders, group chat and message board functions were not implemented. Otherwise, many themes identified in the FGDs were anticipated by the authors and already addressed by features in HBB Prompt v0.

#### Functions and features of the HBB Prompt v0 and modifications based on Phase 1a

**Fig. 3:**
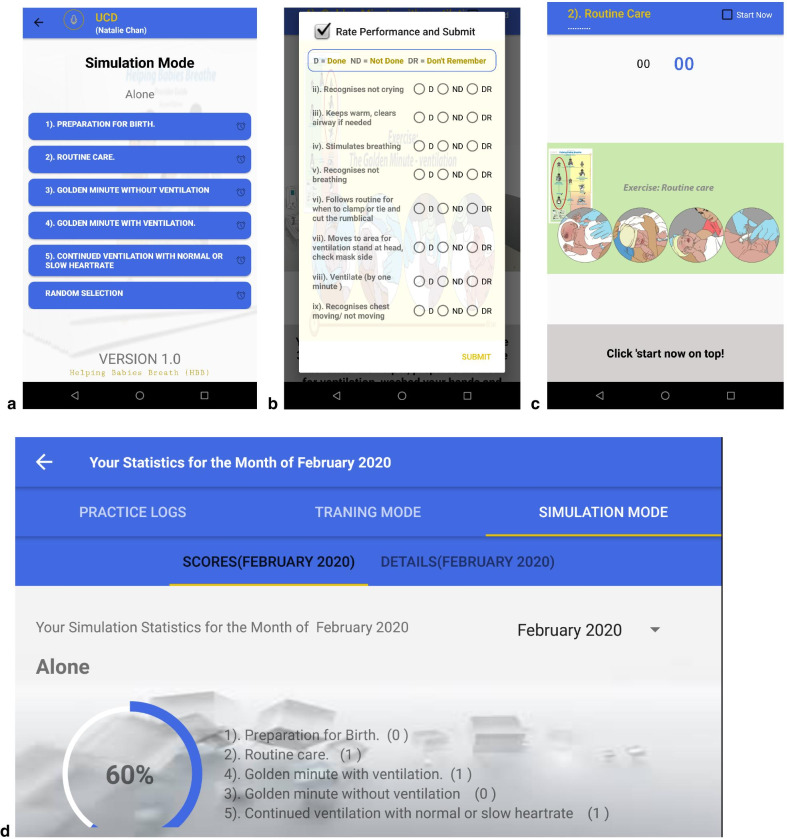
HBB Prompt v1.0 screenshots. **a** different scenarios users can choose from in Simulation Mode. **b** Performance checklist that appears once a simulation is complete. **c** Beginning of a simulation. Note the two countdown timers from the time the simulation begins and the time when the simulated infant is born. **d** Sample of dashboard statistics

Figure 3 shows screen shots of HBB Prompt v1 and Table 3 describes the features of HBB Prompt. **Training mode** was designed to demonstrate each step of the HBB algorithm through videos, aimed as a resource for refreshing knowledge and skills. In response to concerns about lack of equipment, a video demonstrating how to reprocess and maintain equipment was added. Other feedback from Phase 1a helped inform the video content, such as breaking down skills into digestible steps and troubleshooting when effective ventilation is not being achieved.

**Table 3:** HBB Prompt app features during Phase 1b

App function	Description	Rationale for inclusion
Training videos	Series of training videos outlining the steps in HBB (Preparation for birth, Routine care, Golden Minute without ventilation, Golden Minute with ventilation, Golden Minute with normal or slow heart rate)	Videos to serve as review materials, with ability to visualize skills that are being demonstrated
Simulation mode—alone mode	Option of targeting practice to a specific scenario that an individual user chooses versus a random scenario that is chosen from the pool of standard scenarios (Preparation for birth, Routine care, Golden Minute without ventilation, Golden Minute with ventilation, Golden Minute with normal or slow heart rate) Audio and visual cues that accompany the scenario that are timed to simulate an actual delivery	To help prompt users through simulated delivery scenarios to facilitate in context practice of skills
Simulation mode—rater mode	Same as alone mode except that the designated rater has a checklist available to review	To help prompt an observer to assess how well the user completes steps in the HBB algorithm in response to the prompts provided about the baby’s condition To help facilitate team practice and peer-to-peer learning of HBB skills and to assist the rater to systematically give targeted feedback
Quizzes/Knowledge Check	Sets of quizzes with questions generated from the HBB curriculum	To help reinforce knowledge pertaining to the steps and reasoning for actions within the HBB algorithm To address the fear of forgetting knowledge learned after initial training
Scoreboard/Dashboard	Visual tracker providing trends regarding practice frequency and knowledge assessment scores	Information to serve as an external motivator to practise regularly and perform as well if not better than peers To increase accountability for practising and increase confidence in resuscitation skills with increased practice

Simulation mode was designed to facilitate routine practice of HBB skills, running through various scenarios that emphasize different aspects of resuscitation. Simulation mode was divided into alone mode (for individual practice) and rater mode (for group practice or peer-to-peer learning). This feature encourages and supports LDHF training. Consistent practice and increased familiarity and confidence in carrying out the steps of the Golden Minute enable HBB providers to perform all actions within one minute of a baby’s birth. To support this, timer prompts were built into simulation mode to facilitate practice of the Golden Minute. Furthermore, checklists were added at the end of every simulation to help users debrief what went well and what could be improved.

The Quizzes and knowledge check section was designed to help refresh and consolidate knowledge about the HBB algorithm and rationale for steps in resuscitation, providing real-time feedback regarding information retention.

The Scoreboard/Dashboard was created to increase accountability and motivation, encourage all users to practise HBB consistently, and foster confidence by displaying personal performance benchmarks.

### Phase 1b

Table 4 summarizes the feedback that was received during Phase 1b and outlines modifications that were made, where possible, in response to the suggestions from the FGDs. Feedback about HBB Prompt was generally positive and navigation was felt to be overall intuitive. There were two iterations of Phase 1b (one with FGD1 and the other with FGD2). After suggestions were incorporated, the team was satisfied that the HBB Prompt app was ready for deployment in Phase 2 for pilot testing.

**Table 4:** Summary of feedback for HBB Prompt during Phase 1b

Feedback	Modifications made	Selected quotes
*Likes*		
Content flows well and is consistent with the HBB curriculum Can complement use of the HBB flip chart during HBB training Can serve well as a refresher training program	Included additional practical recommendations, such as, when to refer infants for advanced care, how to cut and trim the cord length, how to keep the baby warm	“You can use it on a daily basis and at any time. You don’t have to wait for a workshop, or organized day.” “I am satisfied that this is a dream come true. The last time we met, we had ideas but did not know how that would be incorporated in an app, and I am hoping the developers carry it farther, and avail it as quickly as they could.”
Training videos comprehensive, realistic and highlighted relevant materials from HBB	Supplemented training videos with text to facilitate review	
Demonstration in video of preparation for birth and testing of equipment in front of family—helps promote transparency and confidence in newborn resuscitation process	Kept this video as a part of the app to help encourage this process of transparency	
Quizzes were useful for review, gave immediate feedback and can be useful tool for teaching Random order of questions helped users not relying on memory alone	Some technical flaws noted that were fixed, for example unsaved answers and needing to redo the entire quiz when users tapped the screen in any incorrect place Added a review feature where answers that are correct and answers that were marked are shown to provide feedback to users	
Simulation mode was helpful for self-practice and group practice Audio and visual prompts were effective	Fixed flaws in orientation of images and timing of prompts	“Yes, it will help us practice with our colleagues. For instance, if you are helping someone who does not know, the video guides you and helps you know what do to next, if you forget. It also helps to time you.” “I think the videos helped us, because it directs you on what to do. Because if they kept saying the baby is not breathing, you would know what to do and keep doing it, ‘till the baby breathes.”
*Dislikes*		
Lack of consistency throughout the app (e.g. how prompts are given, audio for crying, where the baby is being resuscitated in the videos)	Modified videos and simulations to use the same prompts In the HBB curriculum timing of cutting the cord, and location of where the baby should be during ventilation (near mother, or on a separate surface) is left up to the discretion of the facilitators teaching in their own setting. For simplicity, decision made to only show ventilation near the mother rather than specifying location depending on the status of the baby	
Some aspects of the training videos were not as realistic, such as the timing with certain actions being too slow	Modified videos to be more realistic and modified timing of transitions in simulation mode	
Font size and menu options at times not clear	Modified text size and colour scheme to improve visibility	
Navigation—suggestion for a restart button in simulation mode	Added pause button (restart feature with back button)	
*General comments and suggestions*		
Using the same HBB curriculum colour scheme of Green, Yellow and Red may help reinforce learning by means of a visual prompt	Added HBB colour scheme in different parts of HBB Prompt content	
For training videos different viewing angles to better capture the details of the actions (e.g. mask seal in bagging)	Decision made not to re-film the videos due to time constraints in filming and editing	
For the Dashboard, use of real names vs. aliases was debated	Consensus was to use real names	
*Customizability*		
Consideration of creating animated videos rather than filming individuals—may allow more flexibility if the app scales to different settings	Not feasible to create animations to address this need within the study time line available	
Including locally relevant practices even if it is not a part of the standard HBB protocol—e.g. asking birth attendants to have bed sheets available for baby in the preparation for birth section as this is common place throughout Uganda, but may not be in other settings	Decision made not to add customized recommendations and adhere to what is presented in the HBB curriculum	

## Discussion

In Phase 1 of this study, we applied a UCD framework and engaged a group of HBB trainers and providers to develop a mobile app, HBB Prompt, to improve knowledge and skills retention after initial HBB training. Many facilitators and barriers identified were feasible to address with HBB Prompt and reflected anticipated features based on prior knowledge about contributors to skills decay. Participants expressed positive perceptions of the app’s ability to help users practise and maintain HBB knowledge and skills.

### Strengths

HBB Prompt is a novel mobile app that facilitates simulation of newborn resuscitation to promote deliberate practice of applying the HBB algorithm to real-life scenarios. We employed a cooperative design UCD framework, which prioritized participation from frontline end-users, both trainers and learners of HBB, since failure of adoption of healthcare technology has been attributed to absence of end-user input [40]. There are different methods to involve end-users in the design and evaluation process, with prototyping and simulation being commonly used in design, and focus groups and observations being common evaluative methods [41]. Understanding the usability of a mobile app and how it meets needs and addresses gaps to improve outcomes have been key to the successful adoption of health technologies. UCD and understanding human factors is crucial since certain ideas may not translate well into a mobile app. Understanding the needs of primary users by exploring acquisition and maintenance of skills and knowledge helped map out areas where HBB Prompt can assist and remaining gaps that need to be addressed through other means [40]. Each function of HBB Prompt was vetted during Phase 1b and largely addressed the issues that were uncovered during Phase 1a.

By iterative testing through participatory design and evaluation, we hoped to overcome the common barrier of failed interventions due to lack of user input. Since the main goal of designing HBB Prompt is to improve knowledge and skills retention after initial HBB training, the UCD process specifically focused on HBB trainer and trainee experiences regarding the process of learning HBB skills and the daily challenges of implementing those skills. We used FGDs to better understand when different cognitive processes took place, influencing our strategies for knowledge and skills retention, a method supported by the applied cognitive task analysis [42]. This strategy has been successful in developing apps for various purposes in healthcare [25, 43, 44], including those in the maternal newborn health domain [29, 32–34, 42].

Newborn health apps [28, 34] have been developed to support HBB training by provision of HBB reference material and clinical decision support. HBB Prompt builds on that foundation and adds new features including Simulation mode and the Scoreboard/Dashboard, which aim to promote LDHF practice. NeoTree [29] is a mobile application developed in a single neonatal unit in Malawi using similar methodology as HBB Prompt, but was intended for data capture of neonatal admissions and clinical decision support. The authors used focus groups to better understand acceptability and feasibility of mobile health (mHealth) solutions before and after implementation of NeoTree. The positive impact of the NeoTree app was attributed to UCD and coproduction. HBB Prompt, on the other hand, applies UCD to create and fine-tune the features deployed in the app. Finally, HBB Prompt builds on the success of the Safe Delivery App (SDA) [30]. The SDA [30] contains education material and videos based on the United Nations Basic Emergency Obstetric and Neonatal Care program (BEMONC) and was evaluated in a large cluster-randomized trial in Ethiopia, which found improved knowledge and skills retention and a statistically non-significant 24% reduction in perinatal mortality. HBB Prompt shares many features with SDA, including video and text as educational materials. HBB Prompt goes beyond the reference tool function by supporting simulation-based skills practice, an interactive feature developed with UCD based on FGD themes of the importance of simulation and peer-to-peer learning. Furthermore, HBB Prompt contains a knowledge assessment feature, which was not available in the early version of the SDA that was tested in the cluster-randomized trial.

### Facilitating simulation

Our app design was informed by the principles of deliberate practice, a well-established methodology for simulation-based medical education [17]. HBB Prompt differs from available apps because it facilitates hands-on practice and simulation, which is a core component of the HBB 2.0 curriculum. While simulated resuscitation will differ from real-life scenarios, consistent practice through simulation is an effective method to maintain competency in managing high-acuity low-occurrence events, such as apnea in a newborn [16, 38]. Much of HBB Prompt’s design focus was to facilitate users’ ability to practise HBB skills in short, directed ways, either alone or with peers. LDHF practice of specific skills such as bag-mask ventilation has been shown to be effective in enhancing skills retention after training [10, 38, 45, 46]. For individuals who are unable practise skills with others, the Training mode videos serve as a guide, and the function of Simulation—alone mode provides a structure and framework whereby users can benchmark their skills and receive automated external prompts and feedback in order to retain their resuscitation skills.

### Use of motivation/behaviour change strategies

The Scoreboard/Dashboard feature was designed to track trends in scores on quizzes, and participants felt it would foster confidence in knowledge retention and increase awareness of knowledge gaps. Being able to visualize frequency and practice patterns serves to increase ownership and accountability for practising as it is a visual audit, providing feedback to each user about habits of practice and where skills may be adequate or lacking [47, 48]. Besides having visual statistics that may benefit end-users, this feature was built to also track app function usage to provide insight in Phase 2 of the study to improve the app and assess which parts of the HBB 2.0 curriculum require more reinforcement. Benchmarking to help healthcare providers inform behaviour change is supported by Social Comparison Theory and Reference Group Theory and has been shown to be a successful strategy to motivate behaviour change [48]. The display of longitudinal trends provides ongoing feedback and is supported by the Feedback Intervention Theory [48]. The Scoreboard/Dashboard feature is unique to HBB Prompt compared with other apps in the newborn mHealth space and evaluating its effectiveness during Phase 2 will further enhance understanding of how audit and feedback can increase retention of knowledge and skills after training.

### Limitations

Although we recruited a diverse group of participants, we may have missed some perspectives. One limitation is the absence of larger scale beta-testing prior to deployment in Phase 2. For example, Bucher et al. invited a large group of users to download and beta-test their app, and provide feedback through a UCD questionnaire [34]. However, the size of our FGDs allowed in-depth exploration and likely uncovered input to reach theme saturation. HBB Prompt’s user interface and functions may be less intuitive for potential users who are less familiar with use of technology as all but one participant (in the HBB provider group) owned a smart device and described themselves as comfortable with their use. Even though the target audience for HBB Prompt are those who were previously HBB trained, including input from first-time learners of the curriculum could have provided additional insight to the knowledge and skills acquisition and retention process. There was an intention to recruit a third focus group with naïve HBB learners; however, due to logistical constraints, we were unable to run a separate series of focus groups with pre-service trainees (medical students and nursing students). On the other hand, our in-depth FGDs solicited input from two groups of HBB users with various experiences that are representative of HBB providers in Uganda, where there is a national mandate for all frontline providers to be trained in both HBB and ECEB. Participant experiences, perceptions, and input through the UCD process were likely generalizable to HBB providers in Uganda and translatable to similar resource settings. Recent clinical and simulation experience were similarly variable across both HBB facilitators and HBB providers, which allowed us to capture opinions that would reflect those with differential exposure to deliveries requiring HBB skills.

While our goal of soliciting input from different types of end-users uncovered many themes that were feasible to address with a mobile app, there are additional limitations to consider. App features addressed intrinsic factors such as motivation and confidence, but not health system constraints, which were frequently cited as barriers. Another challenge of UCD is balancing implementation of suggestions and prolonging the timeline of app development. For example, although Simulation—rater mode was felt to work well, having the ability to link two devices so that the user and the rater could engage in the simulation on different screens would have further enhanced its function but this was technically not feasible given the time constraint of the study. Although customization was requested during feedback, it was important to consider how to be specific enough without reducing generalizability. Keeping the app content close to the HBB curriculum would allow it to scale to other settings more easily.

## Conclusion

With the ubiquity of mobile devices, apps that are carefully designed and evaluated have the potential to scale rapidly and reach a broad audience to effect large scale change. HBB Prompt was developed with UCD input from HBB trainers and providers to tailor its design to support knowledge and skills maintenance after HBB training. HBB Prompt features were built to address themes uncovered, which included motivation to practise, confidence in knowledge or skills, importance of feedback and peer-to-peer learning. The iterative process of incorporating feedback further improved the features and functions of HBB Prompt as a practice tool to facilitate skills and knowledge retention. Pilot-testing of HBB Prompt in Phase 2 of this study will further evaluate the ability of this UCD process to build a mobile tool to address the important issue of increasing sustainability of HBB training.

## Supplementary Information

**Additional file 1.** HBB Prompt Phase 1a Focus Group Guide Plan and Standard Operating Procedures. Focus group guide with operational details, questions and prompts used to conduct focus group discussions during Phase 1a.

**Additional file 2.** HBB Prompt Phase 1b Focus Group Guide Plan and Standard Operating Procedures. Focus group guide with operational details, questions and prompts used to conduct focus group discussions during Phase 1b.

## Data Availability

The datasets used and analyzed during the current study are available from the corresponding author on reasonable request.

## Abbreviations

AAP: American Academy of Pediatrics
app: Application
BEMONC: Basic Emergency Obstetric and Neonatal Care
CEMONC: Comprehensive Emergency Maternal and Newborn Care
csv: Comma-separated values
ECEB: Essential Care for Every Baby
ECSB: Essential Care for Small Babies
ETAT: Emergency Triage and Treatment
FGD: Focus group discussions
HBB: Helping Babies Breathe
IMEI: International Mobile Equipment Identity
LDHF: Low-dose high frequency
mHealth: Mobile health
MUST: Mbarara University of Science and Technology
REC: Research Ethics Committee
REB: Research Ethics Board
SDA: Safe Delivery App
UCD: User centred design
v0: Version 0
