# Supplementary material for: Development of a novel mobile application, HBB Prompt, with human factors and user-centred design for Helping Babies Breathe skills retention in Uganda
Source: BMC Med Inform Decis Mak. 2021 Feb 4;21:39. doi: 10.1186/s12911-021-01406-z (PMC7863544; doi:10.1186/s12911-021-01406-z)
Supplement: Supplementary file 1 — Additional file 1. HBB Prompt Phase 1a Focus Group Guide Plan and Standard Operating Procedures. Focus group guide with operational details, questions and prompts used to conduct focus group discussions during Phase 1a. [file 12911_2021_1406_MOESM1_ESM.docx]

## Additional file 1: HBB Prompt Phase 1a Focus Group Guide Plan and Standard Operating Procedures

**HBB Prompt Team Roles:**

- Timekeeper
- Voice recorder and tablet monitors
- Photographer of flip-chart after each activity segment
- Flip-chart organizer
- Discussion facilitators

**Registration (30 minute period)**

Prep: provide all participants with pens and folder with: agenda, consent form, and demographics form

Setup: registration table with sign-in sheet (accountability sheet with Name, Health Facility, Contact Information), masking tape with marker

- Have each participant register with the sign-in sheet
- Assign each participant a participant ID number (starting with 100)* and write their ID number on a masking tape and have them tape it on themselves in a visible location
- Provide each participant with their folder (containing agenda, consent form, demographics form) and ask them to review the information as they wait for the day to begin
- Provide each participant with a pen they will use throughout the day

**NB – facilitators should assign themselves a number ID beginning with 701

**Introduction and consent process (15 minutes)**

- Welcome and thank participants for coming
- Explain consent form and overview of the day
- Participants to have 10 minutes to review consent form and fill out demographics form if they wish to participate

**Start with icebreaker (10 minutes)**

Prep: blank papers for all participants and pens/pencils for drawing

Setup: participants seated in roundtable

Audio recording: no

- Ask participants to use 3 images to describe their favourite food
- Ask each person to pass their images to the left
  - Go around in a circle and have that individual who received the images describes to the rest of the group what those images mean

**Introduction focus group questions: (20 minutes)**

Prep: voice recorder on table and backup recording on IV pole mounted tablet

Setup: participants seated in roundtable

Audio recording: yes

|  | - Introduction to FGD – Ask participants to identify themselves at the beginning with their participant ID (given to them on a masking tape they will wear) and to please do this every time a recording is started |
| --- | --- |
|  | - - Provide example of how it works: e.g. “701: I think bagging is very hard” “702: I disagree, I think keeping a baby warm is harder” |
| 10-15 min | - Please share some of your personal experiences regarding successes and challenges in stabilization of the newborn |
| 5 min | - Have you participated in any HBB refresher courses? Do you, or would you find them useful? |

**First activity: Barriers/facilitators to learning (~ 1 hour)**

Prep:

- Distribute post-its and pens for participants
- Flip chart / manila sheets posted throughout room with discussion points
- Voice recorder for each station recording – assign each one to one facilitator to manage
- Voice recorder on table and backup recording on IV pole mounted tablet for group discussion

Setup: participants to walk around flip chart stations throughout the room, each station with voice recorder on

Audio recording: yes

Explain activity:

- There are questions and prompts regarding different aspects of HBB learning. We have given each of you a stack of post-its. Please write one thought or idea per post-it and stick them on the paper underneath each question. You may use as many post-its as you like and we would like you to put at least 1 post-it per question. There is no right or wrong answer. You will have **15 minutes** to do this individually, and then we will come together to discuss your ideas as a group.

Start activity:

- Participants walk around and write ideas down on post-its and stick them to flip-charts at each station:
- STATIONS (each bullet = 1 flipchart)
  - If you compare what you thought you needed to know vs. what you actually need to know now, how does it compare?
  - What is most complicated to know now?
  - What took the longest to learn?
  - What are barriers to learning HBB skills?
  - What helps you learn HBB skills?
- Set-up voice-recorder at table. After 15 minutes, ask participants to come back to sit together in roundtable. Remind participants that discussion will be audio-recorded, and to start with identifying themselves by their participant IDs
- Help lead discussion through these questions, and collate helping collate post-its into themes on the flip-chart. Summarize themes once organized and discussion is wrapping up for each question. (45 minutes)

**Second activity: Timeline ~ 1.5 hours**

Prep:

- Distribute more post-its to participants
- Change out flip chart / manila sheets from previous activity
- Organize flip charts with timelines throughout room in stations for pairs
- Voice recorder for each station recording – assign each one to one facilitator to manage
- Voice recorder on table and backup recording on IV pole mounted tablet for group discussion

Setup: participants to form pairs around flip chart stations throughout the room, each station with voice recorder on

Audio recording: yes

Explain activity:

- Please form pairs and bring your post-its towards one of the stations with the timeline flip-charts. This timeline is divided into: before learning the HBB skills, during HBB training, immediately after learning HBB and 3 months after BBB training

For each part of the timeline, there are different questions or factors to address including:

- - - Fears/concerns/perspectives
    - Major learnings
    - What they thought would be the hardest to learn/remember
    - What was actually the hardest to learn/remember
    - What they would have changed to improve the learning situation
- As you write thoughts on post-its in pairs, your discussion will be recorded by the tablet at your stations.
- After working in pairs for about 30 minutes, each pair will present to the rest of the group what is on their timeline.
- After that, we will come together and put our collective thoughts into a final timeline

Start activity

- Start recording with participant IDs and timeline activity
- Facilitators will walk around and listen in and help facilitate and probe discussion
- **Stop pair audio recordings and start group voice recording with tablet backup**
- Everyone comes together and each pair brings their timelines closer and describes their timeline
- Facilitators guide the group as they reflect on what everyone shows in their timelines
- Finally, everyone will come together and put all their thoughts into a final timeline on the wall
- Participants may re-use post-its from their own timelines
- **change out flip chart to group timeline flip chart**
  - Ask for a volunteer or assign one person to lead the creation of a group timeline

**Third Activity: Sketching ~ 1.5 hours**

Prep:

- Prepare handouts and distribute barriers one first
- Voice recorder on table and backup recording on IV pole mounted tablet for group discussion

Setup: participants to form different pairs and to find an area where they want to sit and work on their handouts

Audio recording: yes - When working as pairs, no need to record but when come to group discussion, turn on voice recording with tablet backup

Explain activity:

- Please form pairs with a new partner to work together on this next activity.
- We will provide you with a sequential set of handouts so that you can develop a tool to help learn and maintain HBB skills and address the various discussion points we have had so far today.
- You will have a total of 45 minutes to work in pairs to design this tool and we will provide more handouts every 15 minutes. T
- The handouts are organized so that you can develop different components of your tool to address various challenges you identify in learning and maintaining HBB skills.
- At the end of the activity, you will be asked to present your tool to the group and to explain how your tool works. You will also be asked to show and describe how your tool can address three different challenges in learning or maintaining HBB skills.

Start activity:

- Pairs are formed and provided initial handout on barriers at their work stations
- Participants to be given handouts to sequentially: (15 min intervals)
  - Identify a particular barrier, and how to develop a tool to address it
  - Identify a particular facilitator and how to develop a tool to address it
  - Develop a mobile app with features that would help with HBB skills learning or maintenance
- Facilitators to walk around to listen in and to probe what each pair is developing
- After 45 minutes, pairs will come together in a large group
- **Voice recorder to be turned on for group discussion**
- Each pair will show their tool and demonstrate how different components may work and explain in more detail how their tool will address 3 different challenges in learning or maintaining HBB skills

**Wrap-up**

Prep: voice recorder

Thank all participants and have them reflect on what they learned today and what they feel can be further addressed
