# Supplementary material for: Development of a novel mobile application, HBB Prompt, with human factors and user-centred design for Helping Babies Breathe skills retention in Uganda
Source: BMC Med Inform Decis Mak. 2021 Feb 4;21:39. doi: 10.1186/s12911-021-01406-z (PMC7863544; doi:10.1186/s12911-021-01406-z)
Supplement: Supplementary file 2 — Additional file 2. HBB Prompt Phase 1b Focus Group Guide Plan and Standard Operating Procedures. Focus group guide with operational details, questions and prompts used to conduct focus group discussions during Phase 1b. [file 12911_2021_1406_MOESM2_ESM.docx]

## Additional file 2: HBB Prompt Phase 1b - Focus Group Guide Plan and Standard Operating Procedures

**HBB Prompt Team Roles:**

- Timekeeper
- Voice recorder and tablet monitors
- Equipment set-up - Flip-charts, notepads, HBB equipment/NeoNatalie
- Discussion facilitators
- Note taker

**Registration (30 minute period)**

Prep: provide all participants with a pen and agenda

Setup: registration table with sign-in sheet (accountability sheet with: Name, Health Facility, Contact Information), masking tape with marker

- Have each participant register with the sign-in sheet
- Based on the master participant list with ID numbers from the previous sessions - write their ID number on a masking tape and have them tape it on themselves in a visible location
- Provide each participant with their an agenda and pen

**NB – facilitators should assign themselves a number ID beginning with 701

**Debrief about phase 1a and introduction to phase 1b (10 minutes)**

- Welcome and thank participants for coming back
- Recap last session’s goals: identifying facilitators, barriers and ideas to improve maintenance of HBB skills
- Provide overview of the day
- **Have participants register for an account on HBB Prompt beta**

**Exploration of HBB Prompt beta app (30-50 minutes)**

Prep:

- Provide notepads/paper and pens to all participants

Setup: participants seated in roundtable, may be sharing tablets

Explain activity:

- Have participants freely explore the app in pairs and encourage them to write down their thoughts on their notepads as they go along and “think out loud” – vocalizing their thoughts as they go along – allowing them to talk about each other’s points – building consensus together during the exploration phase
  - What they like and why
  - What they don’t like and why
- HBB team to walk around and write down observations of how users are interacting with the app, specifically what they seem to be having trouble navigating and using and flow in the use of the app

**Exploratory Feedback: (90-125 minutes)**

Prep: voice recorder on table and backup recording on IV pole mounted tablet

Setup: participants seated in roundtable

- Flip chart paper for each of: content, interface, navigation, functionality, customizability, usability

Audio recording: yes

Start activity – unstructured feedback:

| - Recap of how to do FGD – Ask participants to identify themselves at the beginning with their participant ID (given to them on a masking tape they will wear) and to please do this every time a recording is started | |
| --- | --- |
| - - Facilitators to provide example of how it works: e.g. “701: I think bagging is very hard” “702: I disagree, I think keeping a baby warm is harder” | |
| 10-15 min | - Please share your initial thoughts about the app with us. |
| 15-20 min | - What do you like about the app? |
|  | - What is confusing about the app? |
|  | - What are aspects of the app that can be improved? |

Focus on structured feedback regarding all of app design

- Facilitator to go through the following prompts
- Recorder to write down thoughts within each category on flip charts

| 10-15 min | **Content:**   - What do you think about the content that is within the app? What do you think definitely needs to be there/stay? - Is there any missing content you wish were in the app? |
| --- | --- |
| 10-15 min | **Interface:**   - How do you like the organization of the app? - How do you like the flow of the app? |
| 10-15 min | **Navigation:**   - Does moving around in the app meeting your expectations? (Does clicking on the various buttons lead you to what you think should be next?) |
| 10-15 min | **Functionality:**   - Tell us what you think about the features that are in the app (what do you like/not like/what can be improved) |
| 10-15 min | **Customizability:**   - Are there any things you would like to change from the defaults that would tailor to your needs (any user-specific changes to be made available – e.g. font size, colour, screens) |
| 10-15 min | **Usability:**   - Effectiveness – does it meet the goals of helping maintain and practice HBB skills - Efficacy/Efficiency – does it achieve the goals in an efficient, easy manner? If not what can be improved? - Satisfaction – what are you satisfied with? Not satisfied with? |
|  |  |

**Focus on simulation mode (60-80 min)**

Prep:

- NeoNatalie simulators and HBB equipment (3-4) for each pair
- Video recorder(s)

Setup:

- Set up tables with HBB equipment and NeoNatalie
- Set up video recording to record how users interact for simulation mode

Video recording: yes

Explain and demonstrate activity:

- Describe how the purpose of this activity is to provide feedback on the design of alone and rater simulation modes
- Model simulation – alone mode
  - HBB Prompt member can walk through demo of golden minute alone mode – prep NeoNatalie equipment, then prop up the tablet in an appropriate location and start to play.
  - Demonstrate steps of HBB along with the simulation
  - Show how to use the rater checklist

Start simulations with alone mode:

| 15-20 min | - Participants take turns, in their pairs or groups of 3, to try using simulation alone mode - Encourage participants to write down or observe their peers going through alone mode and note what they see as useful and what may be a hindrance / not make sense |
| --- | --- |
| 15-20 min | - Feedback on simulation alone mode in roundtable FGD |

Explain and demonstrate rater mode:

- Model simulation – rater mode
  - Two HBB Prompt team members to walk through set-up screens
  - One team member to demonstrate steps of HBB while the rater makes notes and then uses the tablet to rate performance

Start simulations with rater mode:

| 15-20 min | - Participants in their pairs or small groups to try using simulation rater mode | | |
| --- | --- | --- | --- |
| 15-20 min | - Feedback on simulation rater mode in roundtable FGD - Does the current format and function of the app help them for peer learning? - What do they think about:   - The rating / checklist aspect   - Flow of the simulation and the various screens - What can be improved? | | |
| 10 min | | - Review power point presentation of the different graphics and sounds used in the app and ask participants for their interpretation to see if it’s as we intended, also ask for feedback on how to further improve these |  |

**Focused Feedback through co-discovery: (75-100 minutes)**

Prep:

- Provide notepads/paper and pens to all participants
- Voice recorder(s)

Setup:

- Seated in roundtable for FGD
- Notepads for participants
- Notetaker

Voice recording: yes

Explain and demonstrate activity:

- Participants should review each of the following sections of the app in pairs or groups of 3 and then provide feedback through FGD
- For each topic, allow participants to provide their thoughts, ensuring that the noted topics are covered (facilitator and note taker to keep track)

|  |  |
| --- | --- |
| 15-20 min | - Training mode videos   - Pace, captions, content, visuals |
| 15-20 min | - Quizzes   - Format, content, navigation |
| 15-20 min | - Dashboard   - Content, visuals, customizability |
| 15-20 min | - Time and location of using the app   - Have participants describe where they see the app fitting into a provider’s day   - Any other potential uses of the app? |
| 15-20 min | - Other general feedback |

**Wrap-up**

Prep: voice recorder

- Have them reflect on whether there are any different things we could be doing during the feedback session for the next round
- Thank all participants for their efforts and feedback and ask if there are any last thoughts before the end of the day.
